# Recent evolution of wine exports in a turbulence period: a multiregional input–output analysis

**DOI:** 10.1186/s40100-023-00248-9

**Published:** 2023-03-10

**Authors:** Inmaculada Carrasco, Juan Sebastián Castillo-Valero, Marcos Carchano, Carmen Córcoles

**Affiliations:** 1 Faculty of Economics, University of Castilla-La Mancha, Albacete, Spain; 2 Institute of Regional Development, University of Castilla-La Mancha, Albacete, Spain

**Keywords:** Exports, International trade, Wine, COVID-19, Input–output model, F14, Q17, Q27

## Abstract

This study focuses on analysing the economic impact (in value-added terms) generated by Spanish wine exports during the period 2018–2020, characterized by significant geopolitical changes and market shocks produced by Brexit, the entry into force of the Federal Law on Viticulture and Winemaking in the Russian Federation, the imposition of tariffs by the USA and the COVID-19 pandemic. For this purpose, the multiregional input–output model was used to identify the overall effects (direct and indirect) in different countries deriving from the changes in final demand. The paper shows that both the world wine trade and Spanish wine exports have been negatively affected by the recent changes in the analysed period. It is estimated that the accumulated losses since 2018 as a result of the decrease in the value-added generated by Spanish wine exports exceed 300 million euros. In addition, the results suggest that the negative effects of COVID-19 are greater than the effects caused by the rest of the geopolitical changes.

## Introduction

Spain has maintained a relevant position in the world wine trade since the 1960–1980s, when Spanish wine exports increased drastically because of the fall in domestic demand. From that moment, and until today, a large part of the Spanish production of wine was destined for sale in the international market (Pinilla and Serrano 2008). In this way, Spain joins a highly competitive and globalized market, where it competes with countries of the “Old World of Wine” such as France and Italy mainly, and with countries of the “New World of Wine” such as China, Australia, or the USA. The situation of imbalance between domestic supply and internal demand described for Spain extends to a large part of the main wine-producing countries. As a result, there is an increase in the percentage of world wine production destined for export: according to data from Anderson and Pinilla (2020), in 1980 only 15% of total production was exported, while in 2010 that percentage amounted to more than 40%. This situation, both with the emergence of new producers, has led to intense growth in the international wine trade (Mariani et al. 2012).

Currently, Spain represents an important part of world wine exports, positioning itself as the world’s leading exporter in volume above 2000 million litters, ahead of Italy and France (together represent more than 54% of the total volume of wine exported). Regarding exports in value, Spain occupies the third position, with about 3000 million euros, behind France, and Italy. All data refer to the report carried out by the Spanish Wine Market Observatory (OeMv) regarding the world's main wine exporters as of June 2022. As main importers of Spanish wine we highlight: USA, UK, Germany, Switzerland, Netherlands, Canada, Mexico, France, China, Japan, and Russia.

In this context, it should be highlighted that the wine trade often faces constraints related to both macroeconomic aspects and the different trade policies adopted (Anderson and Wittwer 2018). Specifically, in recent years there have been significant events in the wine industry (changes in the trade regulations of the USA and Russia, Brexit, and the recent COVID-19 health crisis, among others), which, added to the appearance of new producing countries, changes in consumption patterns and direction of trade flows, greater public intervention (Castillo et al. 2016), have affected the volume of wine exports from Spain.

In 2020, the world was rocked by the effects of COVID-19. This massive disaster in the form of a pandemic has had indiscriminate and devastating consequences for people and businesses, varying in relevance depending on the region and sector. To learn from experience, it is important to analyse these consequences in depth in each of them. Despite the proliferation of data on the economic effects of the COVID-19 crisis in different markets, scientific analyses are still required (Genkin and Mikheev 2020).

The crisis caused has challenged food security and agricultural and food systems (Deaton and Deaton 2020; Galanakis 2020). One of the direct effects of COVID-19 has been the disruption of trade between countries (Gray 2020), causing disturbances in demand and weakening agri-food supply chains. Cross-border supply chains are fundamental for the Spanish wine sector. The case of wine is special since it is both an off-premise product and a commodity that is also consumed in social and tourist or leisure activities, all of them severely damaged by the closures and social distancing measures (Compés et al. 2022; Dubois et al. 2021).

In the case of international wine markets, the effects of the pandemic are intertwined with others, which were, by themselves, already capable of having a decisive impact and which have occurred over a decade marked by ups and downs characterized by great uncertainty in the face of internal demand problems and high volatility in foreign markets. Thus, coupled with the effects of the outbreak of the economic crisis in 2008, from which the sector in Spain was slowly recovering thanks to a never-before-seen export effort, a new disruptive element appeared with the vote to initiate the UK's exit from the European Union in 2016, which marked the beginning of a new convulsive period in European markets (Anderson and Wittwer 2017, 2018). The foreseeable shocks to international trade, deriving from this new situation, were exacerbated by the imposition of North American tariffs on viticultural products from certain European countries, including Spain, legislative changes affecting wine in Russia, and turbulence in the Chinese market due to the trade dispute with Australia, not to mention local events such as droughts and floods that are becoming increasingly frequent (Wittwer 2020). The culmination has been the shock in demand produced by the harsh economic consequences of the COVID-19 crisis. The results of this series of events have required companies in the sector to be dynamic and adapt their commercial and internationalization strategies in order to maintain viable access to world markets.

Regarding the recent geopolitical changes, previous studies have focused on the analysis of the effects of Brexit, the imposition of tariffs by the USA, the Russian Wine Law. For example, Anderson and Wittwer (2018) estimate the possible effects of Brexit (“hard” and “soft”^1^) on the international wine market. A decline in the level of UK imports is expected because of three main factors: increased import tariffs, loss of purchasing power because of the real depreciation of the pound, and slow revenue growth. Other authors such as Dhingra et al. (2017) or Sampson (2017) obtain similar conclusions. For their part, Del Bianco et al. (2015) investigate the impact of trade barriers on global wine trade, identifying that tariffs negatively affect trade. More recently, Ridley et al. (2022) estimate that the wine trade dispute between the USA and the European Union will cause a decrease in the annual wine trade of 189.7 million dollars. Tariffs thus become a trade barrier by increasing import prices and hindering access to certain markets. For the opposite case, Greear and Muhammad (2021) highlight that the elimination of the Japanese tariff for European wine would mean an increase in Spanish exports worth 8.2 million dollars with respect to the tariff situation. In this sense, the implementation of trade restrictions (tariff and non-tariff measures) can be expected to cause large losses in the wine trade (Mariani and Pomarici 2019).

In this context, the aim of this paper was to determine the total economic effect in terms of value-added generated by Spanish wine exports in the different countries with which it trades during the period 2018–2020 (characterized by the succession of different geopolitical disturbances and shocks that were culminated by COVID-19 crisis). In order to carry out this analysis, the multiregional input–output (MRIO) model was used. This model considers interdependence in the productive activity of the different sectors of the economy broken down by country and sector. This analysis was performed based on monthly data from the Spanish Wine Market Observatory (“Observatorio Español del Mercado de Vino”–“OeMv”—in Spanish) for the years 2018–2020. This study seeks to complete the existing studies that analyse the impact of geopolitical changes and others shocks on the Spanish wine industry.

The article is structured as follows: “The impact of different shocks on the world wine trade and on Spanish wine exports” section presents information on the impact of different shocks (Brexit, the entry into force of the Federal Law on Viticulture and Winemaking in the Russian Federation, the imposition of tariffs by the United States and the COVID-19 pandemic) on the world wine trade and on Spanish wine exports; “Method and data” section summarizes the methodology applied to achieve the objective of the study and details the sources used to obtain the data; “Direct and indirect effects of the evolution of markets for Spanish wine exports in the different countries and sectors” section analyses the results obtained; and “Conclusions” section presents the conclusions.

## The impact of different shocks on the world wine trade and on Spanish wine exports

In recent decades, globalization, together with the liberalization of international trade, has accelerated export growth (Mariani et al. 2012). Specifically, the elimination of trade barriers and the different trade agreements such as the North American Free Trade Agreement (NAFTA) or the Southern Common Market (MERCOSUR). To these facts, we must add the changes experienced in the industry itself: the increase in consumption in countries of northern Europe, North America, and Asia, the decrease in consumption in traditional countries of the “Old World” and the increase in exports from new producing countries such as the USA, Australia, Chile, or Argentina. All this has led to international trade becoming a predominant element within the wine industry. For example, in the last 25 years, while wine production and consumption have remained relatively stable at around 260 million hectolitres and 240 million hectolitres, exports have grown by more than 70% between 1995 and 2020, accounting for a third of world production (OIV 2022).

Regarding the international wine market, Europe continues to be among the main wine-producing and exporting regions in the world, specifically until 2016 the European Union represented 67% of world wine production and 71% of world exports, that is, more than three-quarters of the volume of production, global wine consumption and trade involves Europe (Castillo et al. 2016). In terms of production and export, the first countries in the world are France, Italy, and Spain (all European countries belonging to the “Old World” of wine).

For its part, Spain stands out for its clear exporting profile (Martínez-Carrión and Medina-Albaladejo 2010). Spain has become the leader in exports in volume with 23 million hectolitres in 2021 (OIV 2022). In addition, its ratio between the volume exported and that consumed domestically is 3.1%, which positions it as a great wine-exporting power. Therefore, given the high exposure of this country to international markets, and taking into account recent shocks (changes in the trade regulations of the USA and Russia, Brexit, and the recent COVID-19 health crisis, among others), this section seeks to analyse the different changes and their effects on world trade and the volume of Spanish exports.

### The effect of the COVID-19 on Spanish wine exports

**Fig. 1:**
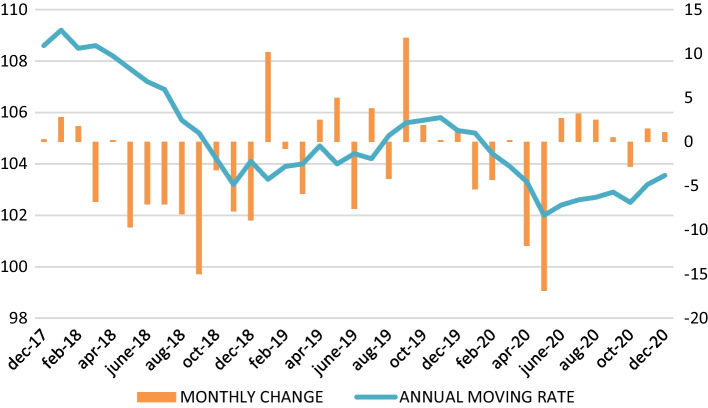
Monthly evolution of world wine exports (million hl). *Source*: Own figure with OeMv data

**Fig. 2:**
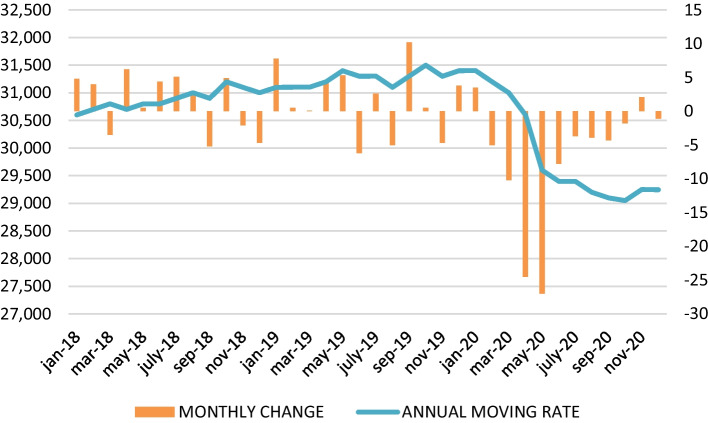
Monthly evolution of world wine exports (€). *Source*: Own figure with OeMv data

COVID-19 has caused a decline in the world wine trade, a market that is often erratic and which tends to show a saw-tooth pattern in its evolution. World wine consumption contracted by 7% in 2020, and a further contraction of an additional 4% is forecast in 2021, while its value fell by 13% in 2020 and is expected to decline by 8% in 2021 (Witter and Anderson 2020). Consequently, the world wine market contracted in 2020, specifically between March and September, with exports falling 1.6% in volume (Fig. 1), representing a loss of 1.63 million hl (OeMv 2021). The same occurred in the value of exports, which fell by 7% (Fig. 2), down 29 cents over the average price (OeMv 2021).

However, the effects of the crisis globally have not been homogeneous and the impacts on the world wine trade have varied, for example, with leading importing countries that have experienced circumstances in parallel to the pandemic crisis (specific details are provided later for Russia, the USA, China or the UK) or also according to types of wine: in the hard semester of the COVID-19 pandemic (March–August 2020), sparkling wines fell 30% in value and 10% in volume, calm wines dropped 17% in value and 8% in volume, while bag-in-box wines increased 4% in value and 14% in volume (Sánchez Recarte 2020). The effect also varied according to distribution channels. In this first semester, the closure of the on-trade channel (open bottle) due to the closure of the HORECA channel was not offset by the increase in volume in the off-trade channel (closed bottle) and above all due to the increase in the bag-in-box channel (OeMv 2022).

### The effect of the China’s policy on Spanish wine exports

In recent years, exports of Spanish wine to China have experienced a negative trend. Specially, 2018 closed with a drop in the volume of − 46.8% compared to 2017, − 5% in 2019 compared to 2018, and − 18.7% in 2020 compared to 2019. The negative trend experienced in 2020 was perhaps linked to the decrease in consumption derived from the COVID-19 crisis (OeMv 2021). In this sense, a report by Wine Intelligence (2020), cited in Witter and Anderson 2020) predicted a 30% slide in the preference for wine in the Chinese market since this is a new product for Chinese consumers and it is mainly consumed outside the home, in social events, which have been more affected by the closures due to the health measures adopted to combat the pandemic.

Although the Chinese market had substantially increased its share of Spanish wine exports in recent years, its imports since 2018 have declined sharply due to the gravitation of its procurements towards Australian wines, which displaced Spanish and Chilean wines (including the bulk segment), even in Tiers 1 and 2 cities,^2^ or in triangulations from Hong Kong.

However, when Australia complained about the origin of COVID-19, China retaliated, thus affecting many imports of agri-food products and especially Australian wines. For its part, the result of investigations into dumping and subsidization (scheduled for August 2021) determined the evolution of temporary tariffs of between 113 and 218% imposed by China on bottled wine imports from Australia, benefiting other producers from Europe and America. Australian exports also experienced several years of drought in important wine-growing regions that reduced the 2019 and 2020 harvests (Witter and Anderson 2020) and were overly dependent on trade with China (Laurenceson and Zhou 2020).

**Fig. 3:**
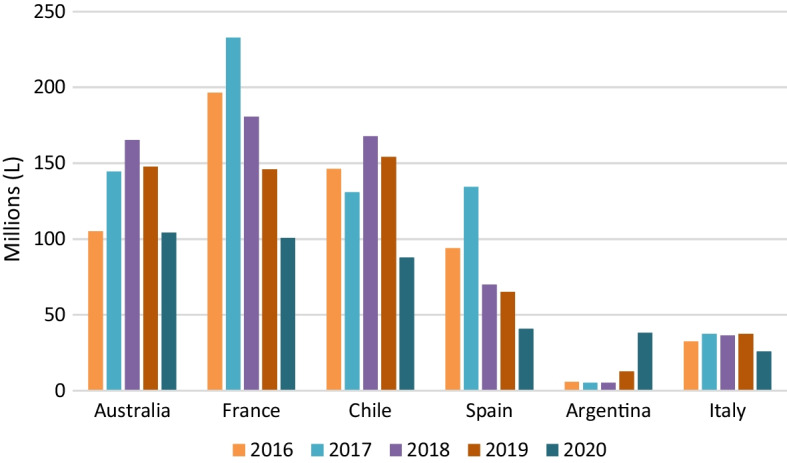
Wine imports in China by country (volume) (2016–2020). *Source*: Own figure with OeMv data

Figure 3 shows the evolution of Chinese wine imports by major supplier countries.

Figure 3 also shows the above-mentioned drop in Chinese wine imports, following several years of decline from the maximum export levels reached in 2017 by France and Spain and in 2018 by Australia and Chile, while Argentine and Italian exports remained very stable. The COVID-19 crisis has had a strong impact on all suppliers, except for Argentina, which in this first year of the pandemic managed to increase substantially the amount of wine sold in China, surpassing Italy, and almost equalling Spain. Australia is now the main supplier but, on a level, very similar to France. It seems that trade in the “Global South” is winning the game over European wines in China.

### The effect of the Brexit on Spanish wine exports

The UK's exit from the European Union (Brexit) and the tough negotiations involved in the said process have also impacted the international wine trade. Brexit must be costly, firstly, for British wine consumers due to the increase in prices due to the imposition of taxes (tariffs). During 2021, numerous bilateral preferential trade agreements (PTAs) were negotiated. The EU-UK trade and cooperation agreement was published in the Official Journal of the European Union on 31 December 2021.

Some authors expected the British wine market to contract, while the trade agreements came into force (Anderson and Wittwer 2018) due to the uncertainty caused in the markets. Therefore, negative effects beyond the initial direct impact were expected.

**Fig. 4:**
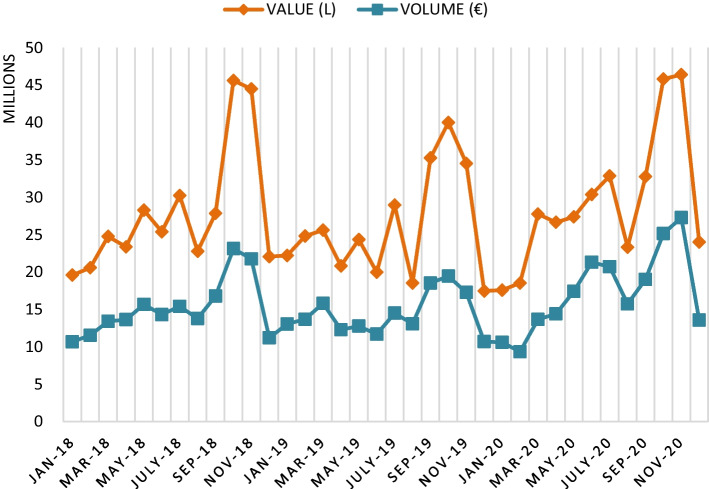
Monthly evolution of Spanish wine exports to the UK (2018–2020). *Source*: Own figure with OeMv data

With the closure of the 2020 campaign, the UK's departure from the European Union has had effects on the wine export markets that have overlapped those induced by the crisis derived from the pandemic. In this case, according to Fig. 4, it is observed that the year 2020 is settled with an increase in Spanish wine exports to the UK, compared to 2018 and 2019. This effect may be driven by supply due to the fear of a hard Brexit (Sánchez Recarte 2020).

Specifically, the volume of Spanish wine exports to the UK fluctuated from − 8.0% in 2018 to − 7.6% in 2019 and + 20.5% in 2020, clearly evidencing the supply effect of importers.

### The effect of the US’s policy on Spanish wine exports

European wine exports to the US market fell during 2020 as a result of the combination of the effects of the crisis deriving from the COVID-19 pandemic and the tariffs imposed by the US government in France, Spain, and Germany for the dispute between Boeing and Airbus.

In October 2019, the Unites Estates administration imposed a 25% value tariff on European wines with an alcoholic strength lower than 14%, as part of the trade dispute. The tariffs were extended until December 2020 to include wines of all alcohol contents. The variation in US wine imports after the first phase of tariffs impacted French wine exports with a decrease of − 12.8% in volume (− 25.3% of value) and − 10.8% in volume (− 13.5% in value) of Spanish exports (OeMv 2021). Italy, as a country not affected by the tariffs, endured the new customs obstacles well. The space left in the US markets by Spanish and French wines was occupied by other suppliers such as Australia.

On 5 March 2021, the European Union and the USA agreed to suspend the retaliatory tariffs for 4 months to focus on resolving the dispute between the two aeronautical companies that has dragged on since 2018. After the announcement, wine buyers returned to the market, with noticeable increases in live bids especially from Bordeaux, followed by Spain, Rhone, and Burgundy.

In this context, it is important not to lose sight of the evolution of the US own production. Californian wines had been affected for some time by overproduction, with a record harvest in 2019 as demand suffered from the adjustments of demographic change (from Baby Boomers to Millenninals). Excess production was reflected in a drop in prices in 2019. The decline in the growth rate of premium wines also became evident, due to greater consumer sensitivity to prices (Bayar 2020).

**Fig. 5:**
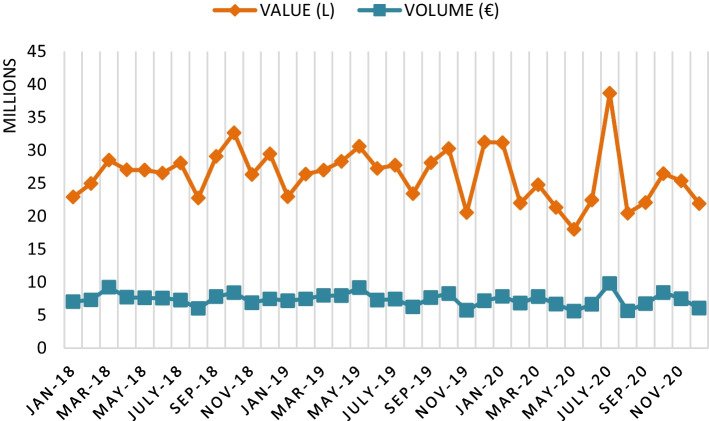
Monthly evolution of Spanish wine exports to the USA (2018–2020). *Source*: Own figure with OeMv data

Figure 5 shows the monthly evolution of wine exports to the USA between 2018 and 2020.

The sum of all these events led to a fall in Spanish wine exports to the USA of − 10% in 2018 (compared to 2017), − 0.8% in 2019 (compared to 2018) and − 4.5% in 2020 (compared to 2019), according to OeMV data.

### The effect of the Russia’s policy on Spanish wine exports

Far from stereotypes, Russian consumers have shown a growing preference for wine, after the recovery from the 2008 crisis, with wine imports growing annually by 13.2% in volume and 20.9% % in value since 2015 (Wine Intelligence 2020), ranking, in 2020, among the top ten most attractive markets in the world (Global Compass Wine Report 2020). This change in preferences is related to the generational change and has been fuelled by the improved availability of wine in Russian markets due to the relative weakness of the euro, the increase in production, especially in geographically close environments such as Europe, the subsequent drop in the wine prices of major exporters and the mainstreaming of trade to Russia among exporters’ objectives.

**Fig. 6:**
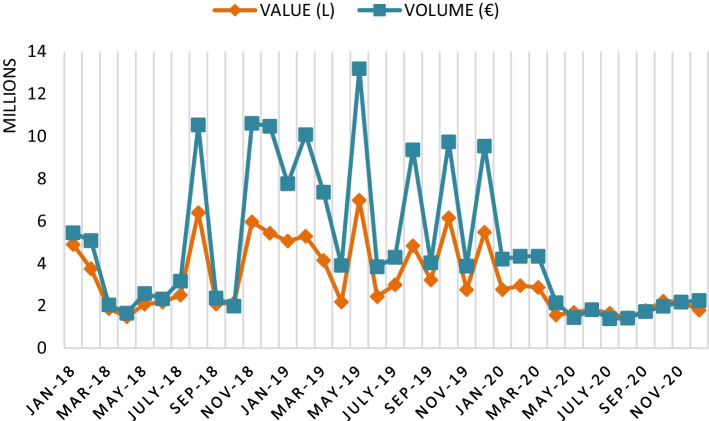
Evolution of exports to Russia by months (2018–2020). *Source*: Own figure with OeMv data

In 2016, Spain was Russia's main wine supplier, with an import market share of 50.49% (OeMv 2021). However, Russia has always been a highly volatile destination for bulk Spanish wine and grape must exports, highly dependent on local harvests from Georgia and other satellite countries. This emerging market is still very small. Therefore, according to OeMv data, the increase in Spanish wine exports to Russia in 2016 and 2017 was followed by a fall of − 45.2% in 2018, an 49.3% increase in 2019 and a further decrease of − 66.4% in 2020. The decline in exports in 2020 reflects the effect of the pandemic together with the entry into force of the new Law on Viticulture and Winemaking of the Russian Federation (Fig. 6).

At the end of 2019, the Russian Parliament approved the Law on Viticulture and Winemaking (LAW 468-FZ), which was created to promote and regulate the growing production of the local industry and represented a signal for investors. The law applies very restrictive definitions of “wine”, “terroir”, “bulk wine”, and “beverages that contain grapes” and may imply the limitation of the qualification of “wine” to products that come from bulk imports. It came into force in June 2020 and the sector expected imports to be negatively impacted and increase in price given the current lack of sufficient local alternatives to substitute imported wines. Bulk wine and grape must imports have been banned (except on rare occasions to enrich alcoholic strength). In the first half of 2020, Russia reduced its bulk wine imports by 55% (those from Spain by 70%) in May and June, and practically no bulk wine was exported from Spain to the Russian market (OeMv 2021).

In this context, the aim of this study was to calculate the direct and indirect economic effects of Spanish wine exports, in recent years, in other countries and sectors. For this purpose, a multiregional input–output model was used. The methodology and results obtained are presented in the following sections.

## Method and data

The method used is based on the multiregional input–output model (MRIO), which is widely used to determine the effects on individual countries and productive sectors of any change in final demand. By using MRIO, we can identify the economic and emissions impacts generated not only in the direct production of goods and services but also those embodied along the whole chain if intermediate inputs (Shapiro 2021). Application and development of methods for estimating these impacts and the elaboration of international databases have allowed deploying this analysis line over the world (Dietzenbacher et al. 2013). Studies based on MRIO to evaluate the impact on value-added (Johnson and Noguera 2012; Koopman et al. 2014), on the job (Simas et al. 2014) or in emissions (Peters 2008), among other impacts, have become leading examples.

MRIO is based on Leontief’s model (1966) and employs the fundamental equation of the latter model to obtain Eq. (1).

1 $$X = \left( {I - A} \right)^{{\left( { - 1} \right)}} Y$$

where *X* is the vector that collects the total output of each productive sector, *A* is the technical coefficients matrix (necessary intermediate consumption per unit of production in each sector), *I* is the identity matrix and, lastly, vector *Y* represents the final demand of the economy (demand on the part of economic agents for each sector).

Equation 2 is expressed in matrix form; this is generalized for *m* regions.

2 $$\left( {\begin{array}{*{20}c} {\begin{array}{*{20}c} {x^{1} } \\ {x^{2} } \\ {...} \\ {x^{m} } \\ \end{array} } \\ \end{array} } \right) = \left( {\begin{array}{*{20}c} {A^{11} } & {A^{12} } & {...} & {A^{1m} } \\ {A^{21} } & {A^{22} } & {...} & {A^{2m} } \\ {...} & {...} & . & {...} \\ {A^{m1} } & {A^{m2} } & {...} & {A^{mm} } \\ \end{array} } \right) * \left( {\begin{array}{*{20}c} {x^{1} } \\ {x^{2} } \\ {...} \\ {x^{m} } \\ \end{array} } \right) + \left( {\begin{array}{*{20}c} {y^{1} } \\ {y^{2} } \\ {...} \\ {y^{m} } \\ \end{array} } \right)$$

In this case, to calculate the economic impact of Spanish wine exports, value-added (VA) was incorporated into the model, which, when divided by total output, gave the value-added (VA) coefficients for each branch of production.

3 $${\text{VA}} = \frac{{{\text{VA}}}}{X}$$

Finally, when performing the operation indicated in Eq. (4), the effect on the value-added generated by Spanish wine exports in the different countries and sectors considered was obtained.

4 $${\text{VA}} = \widehat{va}\left( {I - A} \right)^{{\left( { - 1} \right)}} \hat{y}$$

where $\widehat{va}$ e $\hat{y}$ corresponds to the diagonalized vectors corresponding to the aforementioned vectors va and y.

In terms of the database, the input–output tables corresponding to the latest version published by the World Input–Output Database (WIOD) (Timmer et al. 2015) were used, the last year with available data being 2014 (release 2016), broken down into 43 countries, and a model for the rest of the world, and 56 sectors. WIOD also provides value-added data, while for the value of wine exports in millions of euros at settlement price, data from the Spanish Wine Market Observatory (OeMv 2021) were used. In the calculations, the wine sector was considered in the “manufacture of food products, beverages and tobacco products” sector and its technical coefficients were used. All the calculations performed in this study followed data broken down by sector and region provided by WIOD. However, the results were added to 6 regions or economic blocks to facilitate the presentation of results.

As the years of interest for this study on the economic effects of Spanish wine exports were 2018, 2019, and 2020 and there are no input–output tables available for those years, the last available year was used (2014). This implied the assumption that there has been no change in the technical coefficients of the sector. Following Wood and Meng (2021), our results should not be affected by this assumption because, considering that technological progress takes time, technical coefficients do not vary significantly from 2014 to 2018, 2019, and 2020.

## Direct and indirect effects of the evolution of markets for Spanish wine exports in the different countries and sectors

**Fig. 7:**
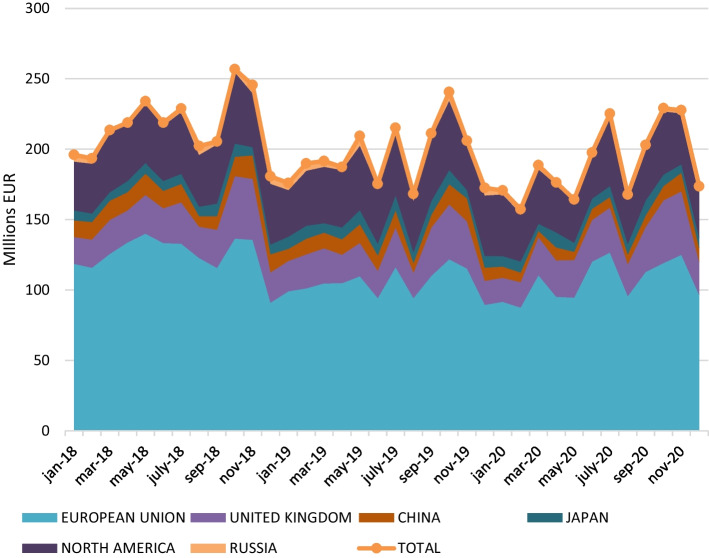
Evolution of value-added (million €) generated by Spanish wine exports (2018–2020). *Source*: own calculation

The application of the model allowed us to obtain the results shown in Fig. 7, presenting the evolution of the value-added generated in the period 2018–2020 by Spanish wine exports in the main economic blocks or countries with which it trades: European Union—27^3^ countries; North America^4^ (USMCA); Russia; UK; China and Japan. This macroeconomic magnitude, which reflects the total added value generated in all sectors of an economic area, reveals a decreasing trend for the entire period studied. Overall, Spanish wine exports generated value-added of € 2594.36 million in 2018, € 2343.20 million in 2019, and € 2281.17 million in 2020 (Tables 1, 2, and 3 in the Annex).

**Table 1:** Impact generated on value-added by country (millions of €) (2018)

	BEL	CAN	CHN	CZE	DEU	DNK	FIN	FRA	GBR	IRL	ITA	JPN	LTU	MEX	NLD	PRT	RUS	SWE	USA	Total
January	6.92	8.41	11.79	1.98	34.81	3.14	1.83	34.56	19.06	1.37	12.38	7.15	2.23	3.96	8.80	6.64	4.76	3.94	22.30	196.02
February	7.78	7.58	12.49	1.15	32.95	4.44	2.13	30.59	20.00	1.16	14.59	6.04	1.20	3.57	9.58	5.80	3.65	4.46	24.29	193.46
March	8.32	8.65	13.64	1.41	34.25	4.93	2.68	34.29	24.08	1.86	11.35	6.02	1.60	5.97	8.48	10.99	1.81	5.50	27.74	213.58
April	9.78	8.34	13.01	1.24	34.69	4.37	2.57	35.57	22.72	1.71	14.91	7.91	2.25	5.24	10.94	10.25	1.44	5.54	26.31	218.80
May	10.92	8.53	15.18	1.26	37.05	5.17	4.60	32.83	27.50	2.54	15.47	7.52	2.42	7.00	10.74	8.26	2.02	8.82	26.27	234.09
June	9.51	7.98	12.39	1.50	33.56	3.83	2.85	37.58	24.69	1.75	13.35	7.04	2.08	5.39	11.10	9.36	2.11	6.90	25.85	218.80
July	11.17	9.32	13.02	1.42	37.25	5.09	3.14	33.77	29.40	2.34	10.42	7.19	2.16	7.23	9.99	10.10	2.44	6.07	27.33	228.86
August	10.62	8.16	7.58	2.67	26.42	3.28	2.27	32.61	22.15	1.42	10.55	6.70	1.50	6.47	9.80	17.23	6.22	4.43	22.15	202.23
September	8.38	8.64	9.69	1.07	33.38	3.46	2.01	31.12	27.09	1.69	6.37	8.88	1.39	5.06	10.56	12.04	2.03	4.26	28.31	205.43
October	10.79	12.68	13.77	1.35	36.60	4.49	3.22	30.10	44.37	2.64	9.61	9.34	2.45	6.27	14.85	13.89	2.09	6.54	31.75	256.80
November	11.63	7.24	16.72	1.45	43.21	4.36	2.58	27.08	43.27	2.98	5.54	5.79	2.97	5.47	13.68	13.23	5.80	6.94	25.61	245.55
December	9.62	7.75	12.82	0.95	25.20	2.47	1.20	20.38	21.46	1.28	5.19	6.98	1.03	6.68	9.23	11.60	5.28	2.95	28.67	180.75
Total	115.44	103.27	152.08	17.46	409.36	49.02	31.08	380.49	325.80	22.75	129.73	86.55	23.28	68.30	127.75	129.39	39.65	66.35	316.58	2594.36

**Table 2:** Impact generated on value-added by country (millions of €) (2019)

	BEL	CAN	CHN	CZE	DEU	DNK	FIN	FRA	GBR	IRL	ITA	JPN	LTU	MEX	NLD	PRT	RUS	SWE	USA	Total
January	7.35	7.25	8.58	0.83	27.63	2.88	1.86	26.93	21.61	1.63	6.30	8.54	1.09	3.58	7.98	9.85	4.92	4.79	22.34	175.93
February	6.52	8.11	11.14	1.18	27.89	3.57	1.77	22.06	24.13	1.36	10.57	9.26	1.14	5.26	9.87	10.14	5.14	5.13	25.67	189.90
March	8.20	9.33	11.05	1.29	30.27	4.66	2.68	24.19	24.90	1.87	5.62	6.74	1.43	4.45	10.03	8.87	4.03	5.66	26.27	191.56
April	8.84	9.02	10.91	1.12	28.17	4.56	2.57	25.61	20.25	1.56	6.18	8.46	1.24	4.14	9.89	9.61	2.12	5.50	27.56	187.32
May	8.53	10.18	13.31	1.23	30.16	5.45	3.30	22.33	23.68	2.05	7.99	10.08	2.19	5.85	11.56	8.35	6.79	6.63	29.77	209.42
June	7.95	8.77	10.86	1.04	23.53	4.27	2.29	21.99	19.42	1.88	5.95	8.34	1.32	4.73	8.92	9.55	2.37	5.66	26.51	175.34
July	9.57	10.03	12.22	1.06	30.00	4.69	2.58	27.38	28.17	2.29	7.33	10.55	2.23	8.04	11.01	10.69	2.91	7.44	26.99	215.19
August	7.98	8.33	6.98	0.78	23.86	2.48	1.65	20.64	18.02	1.54	11.96	7.58	1.43	5.51	8.25	10.26	4.70	3.49	22.79	168.26
September	6.97	10.64	9.07	1.15	35.08	3.65	2.07	24.04	34.29	2.10	10.03	9.82	1.55	6.81	8.62	9.11	3.13	5.70	27.36	211.21
October	10.16	11.81	14.44	1.49	32.38	4.05	2.49	25.88	38.89	2.78	10.56	10.13	1.80	8.05	11.90	12.40	5.99	5.95	29.45	240.60
November	12.24	7.12	16.30	1.86	32.39	3.85	2.15	22.10	33.58	2.85	7.23	5.92	1.65	5.10	13.45	8.54	2.69	6.94	19.99	205.97
December	8.13	7.26	9.39	1.26	21.73	2.90	1.46	20.15	16.99	1.84	5.41	8.28	1.54	5.31	11.36	10.49	5.32	3.28	30.40	172.50
Total	102.44	107.86	134.26	14.29	343.10	47.00	26.85	283.29	303.93	23.76	95.15	103.71	18.61	66.84	122.86	117.88	50.10	66.15	315.11	2343.20

**Table 3:** Impact generated on value-added by country (millions of €) (2020)

	BEL	CAN	CHN	CZE	DEU	DNK	FIN	FRA	GBR	IRL	ITA	JPN	LTU	MEX	NLD	PRT	RUS	SWE	USA	Total
January	6.57	8.51	7.99	1.02	26.94	2.26	1.54	20.89	17.11	1.58	6.77	7.44	0.90	5.03	9.34	9.38	2.69	4.40	30.32	170.68
February	4.95	9.17	6.89	0.79	25.15	2.89	1.76	20.30	18.02	1.72	7.36	7.82	0.89	3.57	7.66	8.53	2.87	5.56	21.40	157.31
March	7.70	9.37	4.50	1.35	36.67	4.70	2.11	23.23	26.99	2.49	5.34	5.03	0.93	5.29	8.99	9.18	2.79	7.86	24.12	188.64
April	5.94	10.84	9.15	1.16	28.17	3.89	2.91	17.23	25.92	2.70	6.55	10.63	0.78	2.38	10.65	9.97	1.52	5.22	20.77	176.38
May	7.07	9.35	5.92	1.05	27.49	4.53	2.36	19.51	26.63	2.19	5.63	6.39	0.96	2.16	10.39	8.64	1.65	4.85	17.54	164.29
June	11.22	6.34	7.91	1.09	26.89	5.92	2.17	25.34	29.55	4.21	5.68	7.20	1.44	2.71	21.03	9.63	1.77	5.57	21.85	197.51
July	11.50	8.56	7.43	1.51	35.25	4.34	2.85	23.86	31.95	2.44	11.65	7.79	1.22	3.61	12.87	11.13	1.60	8.05	37.61	225.20
August	7.49	9.11	6.93	0.98	22.29	3.12	1.73	23.72	22.67	1.96	9.67	7.48	0.91	4.54	9.02	11.25	1.40	3.59	19.91	167.76
September	6.59	11.15	8.20	1.29	30.39	3.62	2.46	26.23	31.86	2.72	11.46	10.54	1.37	5.10	11.27	9.16	1.72	6.38	21.49	202.98
October	8.62	13.84	10.09	1.80	33.08	4.62	2.36	23.34	44.55	3.43	12.10	8.12	1.64	5.40	11.19	9.81	2.15	7.19	25.76	229.09
November	10.29	7.72	13.15	1.88	33.58	5.06	2.27	23.40	45.12	4.50	10.76	6.02	1.45	3.86	13.50	9.64	2.14	8.67	24.67	227.68
December	7.09	9.02	9.96	1.36	27.00	3.06	1.04	18.30	23.36	1.61	7.38	7.42	1.01	4.31	13.29	10.50	1.74	4.86	21.33	173.65
Total	95.03	112.97	98.11	15.28	352.91	48.01	25.57	265.35	343.73	31.56	100.33	91.88	13.50	47.94	139.19	116.80	24.05	72.19	286.78	2281.17

However, stand-out variations can be observed, depending on the disruptions in volume and/or value of Spanish wine exports. Thus, the value-added generated by said exports grew steadily during the first two quarters of 2018, from € 196 million in January to € 228.9 million in July, reaching their maximum level in October of this year (€ 256.8 million). In successive quarters, between October 2018 and August 2019, value-added registered a cumulative decrease of 41%, which has continued until early 2021, 313.19 million euros lower than in the 2018 campaign. It should also be noted that the outbreak of the COVID-19 crisis in November 2019 exacerbated the fall in value-added generated by Spanish wine exports, specifically during the hardest months of the crisis (March, April, May) when these fell by € 58.99 million compared to the same months in the previous year.

The multiregional analysis carried out in this study allowed us to determine the weight of value-added generated in each country studied and the decline experienced between 2018 and 2020. Specifically, in 2018, the 58% of the value-added is generated by Spanish wine exports to the European Union, 18% by exports to North America, 13% by exports to the UK; the rest of the value-added generated is because of exports to China (6%), Japan (3%), and Russia (2%). Consequently, the changes in aggregate value-added are largely expected to be explained by the growth or decrease in value-added generated by wine exports to these blocks or countries. Changes were observed in the weight of each block or country in 2020, concentrated mainly in the European Union (− 3%), China (− 2%), the UK (+3%), and North America (+2%).

**Fig. 8:**
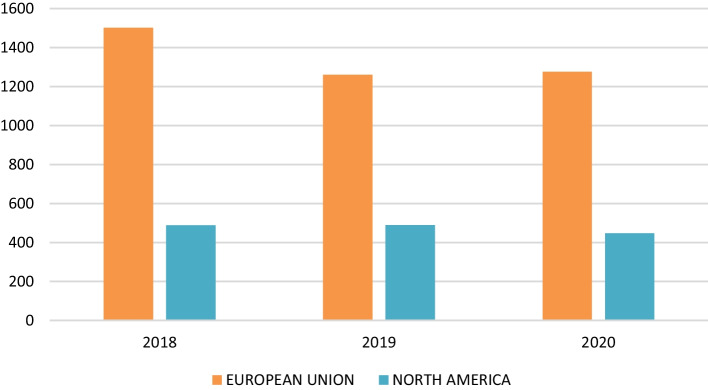
Annual impact generated on value-added by economic blocks. *Source*: own data

Figure 8 shows the value-added generated in the countries because of Spanish wine imports during the analysed period. In the pre-crisis period, the bloc formed by the European Union generated € 1092 million, thanks to the contribution of Germany, France, and Italy, which, respectively, accounted for 37%, 35% and 12% of total value-added generated in this economic block in 2018. Similarly, in North America as a whole, these imports generated € 488 million, with the USA (€ 316 million) being the main contributor, followed by Canada (€ 103 million) and Mexico (€ 68 million). For its part, in the UK Spanish wine imports generated €325 million, ranking in third place behind Germany and France. Finally, in China, Japan, and Russia Spanish wine exports generated € 152 million, € 86 million and € 40 million of value-added, respectively.

**Fig. 9:**
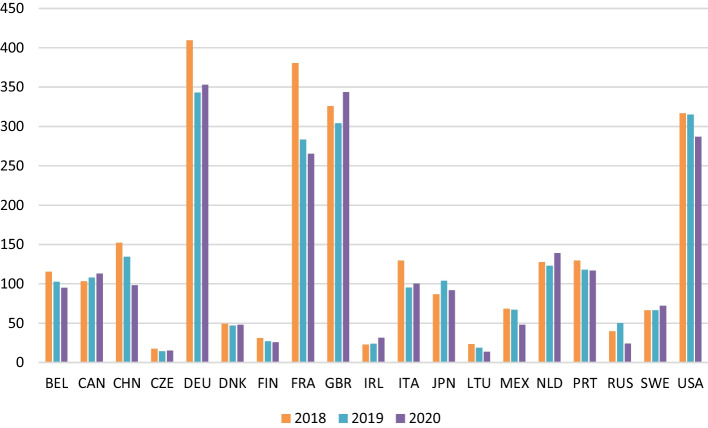
Annual impact generated on value-added by country. *Source*: own calculation

Focusing on the period coinciding with the outbreak of the health crisis caused by COVID-19 (2020) and attending to Figs. 8 and 9, by economic blocks it was observed that € 226 million less was generated in the European Union than in 2018, and € 13 million more than in 2019. The fact that the value-added generated is higher than in 2019 can be explained by the increase in the value of exports, and, therefore, the contribution to value-added of minority countries such as Ireland, the Netherlands, and Sweden, since the other countries in this bloc have accumulated losses amounting to 16 million euros. Similarly, the USMCA registered a decrease in value-added generated in both 2018 and 2019; specifically, € 40 million were lost between these two years. However, North America is a specific case since it is difficult to differentiate the effects of COVID-19 itself from those deriving from the increase in tariffs adopted by the Trump administration; while the USA reduced its share of value-added, with an accumulated loss of 28 million euros between 2019 and 2020, Canada registered an increase of € 10 million euros in the same period. The same occurred with the UK, as its contribution to value-added generated by exports grew by 6% and 13% compared to 2018 and 2019, respectively, mainly as a result of the supply effect caused by the breakdown of trade agreements after Brexit. Of the remaining countries, only Japan contributed positively to value-added during the period, while China and Russia had a negative impact, which, together with the health crisis, may be attributed to the moderate import activity of the former in favour of Spain, and the new wine legislation enacted in Russia penalizing bulk wine imports.

**Fig. 10:**
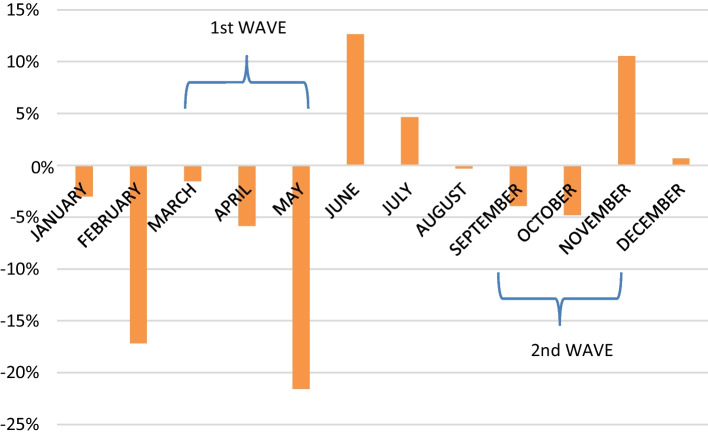
Variation in the effect on the value-added of Spanish wine exports in 2020 with respect to the same month in the previous year (%). *Source*: own source

As announced in the previous chapter and demonstrated previously, other adversities must be considered in addition to the specific effects deriving from the COVID-19 pandemic, namely Brexit, the entry into force of the Law on Viticulture and Winemaking in the Russian Federation, or the tariffs imposed by the US. In this context and focusing on the period in which the economic impact of COVID-19 was greatest, Fig. 10 shows the percentage variation in value-added generated by Spanish wine exports with respect to the same month in the previous year. As can be observed, in February, even before the state of alarm was decreed by the World Health Organization, 33 million euros less were generated than in the same month one year before. This situation worsened during the toughest months of the first wave (April and May) in which € 56 million less was generated than in the same period of the previous year. Since the end of the first wave, and coinciding with the summer months, value-added increased in two successive months (June and July) (+€ 32 million). November and December, together with the summer months, mitigated the losses during the first quarter of 2020, with a positive economic impact on value-added of 55 million euros. By quarters, the second quarter, which coincided with the most critical moment of the crisis, presented the worst results, with an aggregate drop of 10% compared to the same quarter in the previous year. The third quarter was the best, with a slight increase of 7%. In short, it was observed that the decrease in value-added was more intense during the months of confinement.

**Fig. 11:**
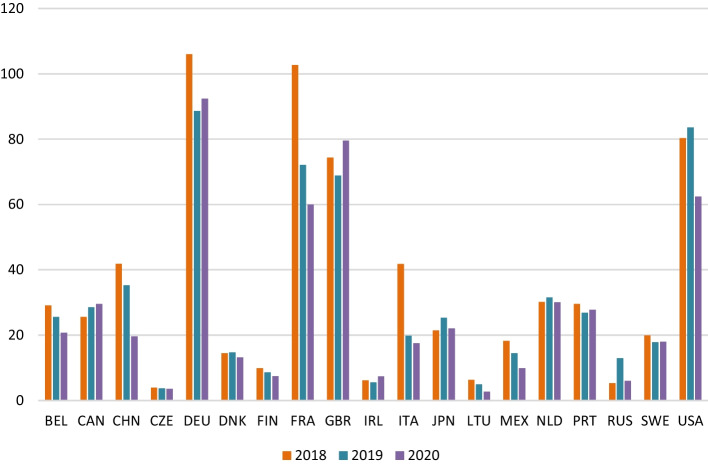
Cumulative impact generated on value-added by country (March, April, May 2018–2019–2020). *Source*: own source

Although it is impossible to separate the effect of the pandemic from the other concurrent factors, Fig. 11 presents the accumulated value-added generated in the different countries for the months of March, April, and May in the three years analysed.

The variation in value-added in the toughest months of the pandemic, compared to the previous year, shows that the first wave of the COVID-19 crisis had a stronger impact on France, Italy, Germany, China, and the USA. The value-added generated in March, April and May fell with respect to the previous year by € 73 million in 2019 and € 47.5 in 2020. Overall, in these countries, the contribution to value-added decreased by 120.7 million euros in those months since 2018, with France, Italy, and Germany recording the sharpest declines of more than 80%, while the decreases in the United States and China amounted to € 17 and € 22 million, respectively. Thus, the European Union is the economic bloc in which the value-added generated by Spanish wine exports fell most during the hardest months of confinement. This contrasts with Canada and the UK, where the global effect with respect to 2019 resulted in increases of € 4 million and € 5 million, respectively.

As said, the crisis deriving from the COVID-19 has overlapped with other events in certain markets. Focusing on the Chinese market, two events overlap, on the one hand, the change in wine consumption patterns in China stands out, towards producing countries of the new world, which translates into a slight fall in the value-added of 2019 of 18 million euros compared to 2018. In addition, the closure of Chinese markets at the end of 2019 derived from the beginning of the pandemic in this country deepened the fall of the value-added for that year, a fall that was maintained in 2020, which closes with a decrease of €36 million (compared to 2019). On the other hand, the approval of the UK’s exit from the European Union inhibits the effects derived from the pandemic. Specifically, the results show that the added value generated by imports of Spanish wine from the UK increases throughout the period analysed because of the supply effect because of the country’s definitive exit from the European Union. The overall effect is summarized in €326 million generated in 2018, €304 million in 2019 and €344 million in 2020. In addition, the entry into force of the tariff imposed by the USA on certain European products, which include Spanish wines, together with the effects derived from COVID-19, affected the volume of wine imports to the USA, which resulted in a decrease in the value-added generated. Despite adverse events, the value-added produced by Spanish wine exports in the USA is estimated at €316 million in 2018; €315 million in 2019 and €287 million in 2020. Finally, it is worth noting the contraction of the export of Spanish wines has also been affected by the entry into force of the new Russian wine law. The value-added generated by exports to that country has been €40 million in 2018; €50 million in 2019 and €24 million in 2020.

Furthermore, focusing exclusively on the first three months of the widespread impact of the virus (March, April, and May), value-added generated by Spanish wine exports decreased by € 89 million, before recovering in the following months. In the 2nd quarter, the figure was 10% down on the same quarter in the previous year. The sharpest decreases were observed in France, Italy, and Germany (more than 80%) and, to a lesser extent, in the USA and China.

## Conclusions

The aim of this work was to calculate the value-added generated by Spanish wine exports in the main export markets, in the reference period. The first conclusion that can be drawn from our calculations is that value-added shows a declining trend. In 2018, value-added tended to grow until October (except in some months such as June and August) and the trend has reversed, with decreases during 2019 and sharper falls in 2020, the year of the COVID-19 crisis. Specifically, the decrease from January 2018 to December 2020 amounted to € 313.19 million. In this period, global wine demand has suffered from the effects of the market contraction in a somewhat turbulent scenario affected by circumstances, such as the UK's exit from the European Union, the imposition of North American tariffs on viticultural products from certain European countries, including Spain, legislative changes in Russia and turmoil in the Chinese market due to the trade dispute with Australia, or the COVID-19 pandemic.

The results suggest that the effects derived from COVID-19 are greater than those caused by the rest of the changes. The COVID-19 pandemic has caused global market contraction. Specifically, the measures adopted by governments around the world to curb the spread of COVID-19 significantly conditioned Spanish wine exports. The closure of bars and restaurants affected wine consumption, which stopped the marketing process, which was not fully compensated by the increase in wine consumption in households.

On the other hand, considering the importance of global value chains in the wine industry, it is expected that the effects analysed will not only be concentrated in Spain and will also be transferred to other regions. Specifically, the effects will be greater in those regions that use Spanish wine as input to produce national wines, or that are simply dedicated to the re-export of Spanish wine.

Assessing the impact and economic effects of the decrease in Spanish wine exports collaborates in the development of appropriate strategies to reduce the sector's vulnerabilities to this type of disturbance and develop appropriate strategies in search of greater resilience of the global supply chain. In this sense, the present study can serve as a starting point to address similar events.

From a managerial perspective, the results have important implications. Managers must bet on strategies such as the search for efficiency through cost reduction, incorporation of process innovations and products that increase customer value, internationalization and allow wineries to access new market niches. In addition, our study highlights the importance of incorporating great diversification of destinations and increase sales in new emerging markets with the maintenance of their market shares in traditional markets. The results are also important for public institutions, which must provide support that encourages innovation, the improvement of managers' digital skills, and even the diversification of markets, products, and segments.

The limitations of the present study are related to the methodology used. As mentioned in “Method and data” section, for the calculations we have used the input–output tables corresponding to the year 2014 (the latest available). In addition, the calculations made do not allow us to differentiate between the specific impact of each of the events mentioned. Further studies are needed to update the calculations made for the Spanish case, since there are currently new databases of input–output tables such as Exiobase that have data up to 2021. In addition, future lines of research should focus on the assessment of the economic impact of the above-mentioned events in other territories and over a wider temporal scope.

## Data Availability

World Input–Output Database (WIOD): https://www.rug.nl/ggdc/valuechain/wiod/wiod-2016-release?lang=en. Ministerio de Agricultura, Pesca y Alimentación (MAPA). Datos de Las Denominaciones de Origen Protegidas de Vinos (DOPs). Available online: https://www.mapa.gob.es/es/alimentacion/temas/calidaddiferenciada/informedops2017-2018modif_tcm30-513739.pdf.

## Annex

See Tables 1, 2, and 3.

## Footnotes

1 Considering that all the commercial agreements that the UK had were centralized through the European Union. A “soft” Brexit refers to a situation in which the UK negotiates and implements free trade agreements after leaving the EU. The opposite situation refers to the “hard” Brexit.

2 The Chinese city tier system is a classification according to size and development. It reflects differences in consumer behaviour, income level, population size, consumer sophistication, business opportunity, among other. Specifically, Tier 1 cities refer at the most developed, large, and densely populated urban metropolises, with important economic, cultural, and political influence in China. Concerning the wine market, Tier 2 cities and lower-tier cities are at an earlier stage in the imported wine market, but they have more growth potential.

3 Includes: Belgium, the Czech Republic, Germany, Denmark, Finland, France, Ireland, Italy, Lithuania, the Netherlands, Portugal, and Sweden.

4 Includes: USA, Canada, and Mexico.
